# Telestration with augmented reality improves surgical performance through gaze guidance

**DOI:** 10.1007/s00464-022-09859-7

**Published:** 2023-01-06

**Authors:** Eleni Amelia Felinska, Thomas Ewald Fuchs, Alexandros Kogkas, Zi-Wei Chen, Benjamin Otto, Karl-Friedrich Kowalewski, Jens Petersen, Beat Peter Müller-Stich, George Mylonas, Felix Nickel

**Affiliations:** 1 Department of General, Visceral and Transplant Surgery, Heidelberg University Hospital, 69120 Heidelberg, Germany; 2 Hamlyn Centre for Robotic Surgery, Imperial College London, London, SW7 2AZ UK; 3 Department of Surgery and Cancer, Faculty of Medicine, Imperial College London, London, SW7 2AZ UK; 4 Department of Urology and Urological Surgery, University Medical Center Mannheim, Heidelberg University, 68167 Mannheim, Germany; 5 Department of Medical Image Computing, German Cancer Research Center, 69120 Heidelberg, Germany

**Keywords:** Laparoscopy, Minimally invasive surgery, Augmented reality, Surgical training, Telestration, Eye tracking, Gaze Guidance, Patient Safety

## Abstract

**Background:**

In minimally invasive surgery (MIS), trainees need to learn how to interpret the operative field displayed on the laparoscopic screen. Experts currently guide trainees mainly verbally during laparoscopic procedures. A newly developed telestration system with augmented reality (iSurgeon) allows the instructor to display hand gestures in real-time on the laparoscopic screen in augmented reality to provide visual expert guidance (telestration). This study analysed the effect of telestration guided instructions on gaze behaviour during MIS training.

**Methods:**

In a randomized-controlled crossover study, 40 MIS naive medical students performed 8 laparoscopic tasks with telestration or with verbal instructions only. Pupil Core eye-tracking glasses were used to capture the instructor’s and trainees’ gazes. Gaze behaviour measures for tasks 1–7 were gaze latency, gaze convergence and collaborative gaze convergence. Performance measures included the number of errors in tasks 1–7 and trainee’s ratings in structured and standardized performance scores in task 8 (ex vivo porcine laparoscopic cholecystectomy).

**Results:**

There was a significant improvement 1–7 on gaze latency [*F*(1,39) = 762.5, *p* < 0.01, *η*_p_^2^ = 0.95], gaze convergence [*F*(1,39) = 482.8, *p* < 0.01, *η*_p_^2^ = 0.93] and collaborative gaze convergence [*F*(1,39) = 408.4, *p* < 0.01, *η*_p_^2^ = 0.91] upon instruction with iSurgeon. The number of errors was significantly lower in tasks 1–7 (0.18 ± 0.56 vs. 1.94 ± 1.80, *p* < 0.01) and the score ratings for laparoscopic cholecystectomy were significantly higher with telestration (global OSATS: 29 ± 2.5 vs. 25 ± 5.5, *p* < 0.01; task-specific OSATS: 60 ± 3 vs. 50 ± 6, *p* < 0.01).

**Conclusions:**

Telestration with augmented reality successfully improved surgical performance. The trainee’s gaze behaviour was improved by reducing the time from instruction to fixation on targets and leading to a higher convergence of the instructor’s and the trainee’s gazes. Also, the convergence of trainee’s gaze and target areas increased with telestration. This confirms augmented reality-based telestration works by means of gaze guidance in MIS and could be used to improve training outcomes.

**Supplementary Information:**

The online version contains supplementary material available at 10.1007/s00464-022-09859-7.

Minimally invasive surgery (MIS) has become the gold standard in many procedures in intraabdominal surgery throughout the last thirty years [1–3]. MIS brings faster postoperative recovery, reduction in postoperative pain, lower rate of surgical site infections and shorter hospital stays [4]. However, MIS also harbours some technical challenges which may result in longer operative times, mostly due to prolonged learning curves [5]. The indirect camera view, the lack of haptic feedback and difficult instrument handling due to the fulcrum and pivoting effects are possible explanations for this phenomenon [6, 7].

To compensate for this prolonged learning curve structured and extensive training of minimally invasive skills is essential. Various box trainers, Virtual or Augmented Reality training systems can significantly decrease the time needed to learn not only basic tasks such as suturing and knot tying but also surgical procedures such as cholecystectomies [8–10]. However, clear and understandable instruction at the training site is still needed to maximize the learning experience of the trainee [11].

To improve the communication between the instructor and the trainee, some innovative programmes and add-ons have been developed lately [12]. Augmented Reality (AR) systems blend artificial images on the MIS screen of the real situs, helping the trainer to point out important structures using arrows, points and sketching over the MIS image [12–14]. A newly developed AR tool called iSurgeon allows using the instructor’s own hand to project gestures in real-time onto the laparoscopic screen and provide visual guidance. Pointing at the target structures or demonstration of the correct execution of the movement becomes effortless and very concise [15–17]. Previous studies have shown that using the system for instruction can lead to time savings and fewer mistakes while performing basic tasks [16, 18, 19].

These results suggest that the changed gaze behaviour of the trainee might play a crucial role in learning and conducting new tasks and it is boosted by AR telestration [12, 18, 19]. An increasing number of studies observing the gaze behaviour of surgeons in laparoscopic surgery and training have shown significant differences in the gaze behaviour of experienced surgeons and surgical novices. [20–22]. AR telestration was able to improve the fixation concentration, gaze latency, gaze convergence and collaborative gaze convergence [12, 18, 19]. Therefore, the aim of the present study was to analyze the effects of AR telestration on the laparoscopic performance of laparoscopically naïve trainees with regard to eye gaze behaviour.

## Materials and methods

### Study design

**Fig. 1:**
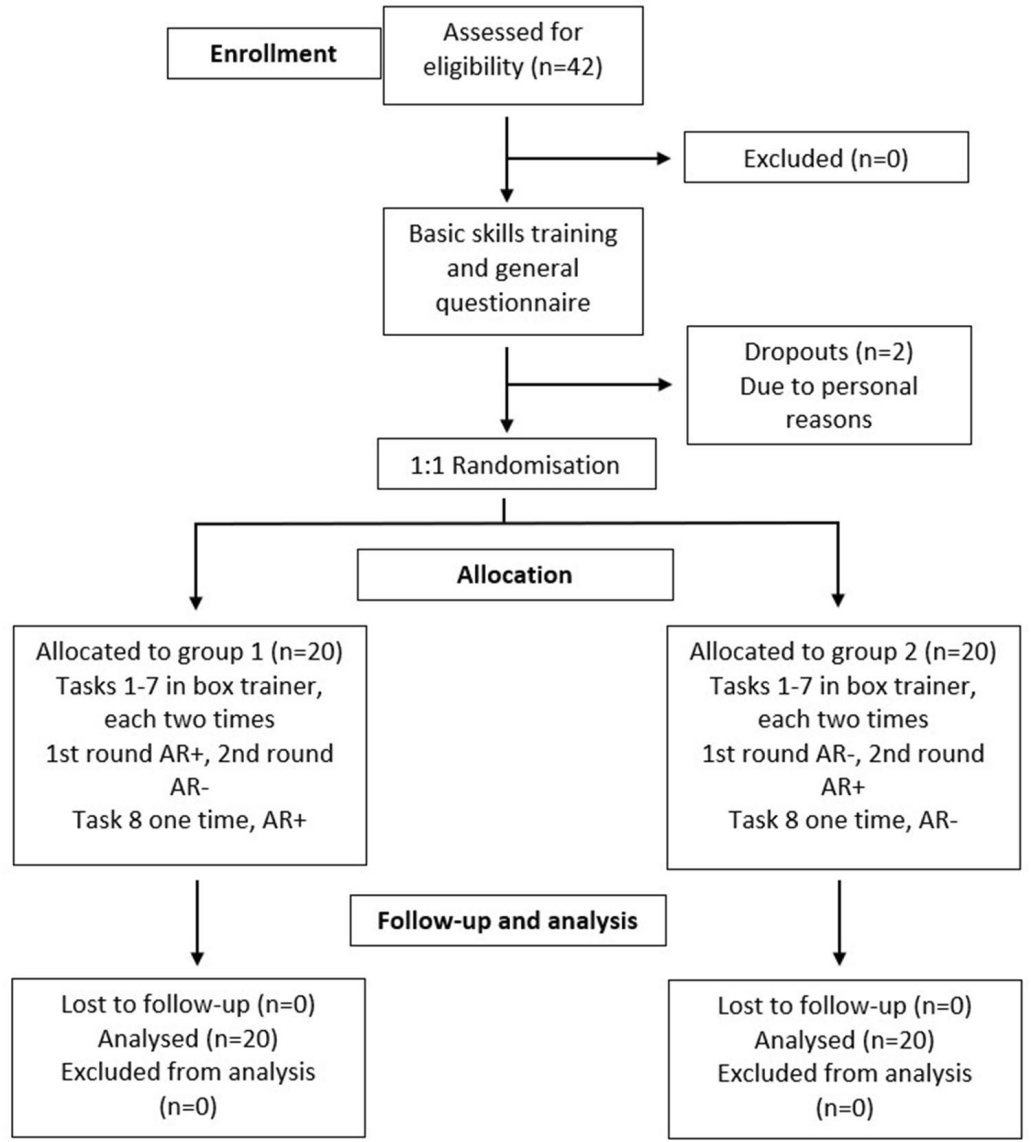
Flow chart of the study design. *AR* + *, *instruction via Augmented Reality telestration with iSurgeon and verbally; *AR*−*, *only verbal instruction

The study was designed as a randomized-controlled, crossover trial with 40 laparoscopically naive medical students recruited through the medical faculty of the University of Heidelberg as part of a clinical elective course. The participants were randomized into two groups (AR telestration and no AR telestration) with an allocation ratio of 1:1 and switched groups after completing all tasks (Fig. 1). A pragmatic sample size was chosen based on the results of previous studies [16].

The participation was on a voluntary basis and all participants signed an informed consent form and filled in the demographic data and surgical experience. The study was performed in the Training Center for Minimally Invasive Surgery at the Department for General, Visceral, and Transplant Surgery at Heidelberg University Hospital, Germany between September 2020 and February 2021. The study was approved by the local ethics committee at Heidelberg University (S-436/2018).

### Laparoscopic tasks

All participants underwent a basic laparoscopic consisting of 6 tasks from the basic module (Task 3–8) on a Virtual Reality (VR) Trainer (LAP Mentor III, 3D Systems, Rock Hill, USA) and two PEG transfers and threading rubber bands through multiple eyelets in a box trainer. Following that, eight different laparoscopic tasks were performed: (1) PEG-Transfer (2) circle marking (3) needle parkour (4) Grabbing and transferring silicone loops (5) Unravelling small intestine convolute (6) blood vessel ligation (7) felt cloth exposition (8) cholecystectomy in a cadaveric porcine liver (Supplementary Table 1; Supplementary Fig. 1). The tasks were selected based on previous studies to investigate the effects of AR instruction in diverse environments [16, 23]. Tasks 1–7 were considered basic tasks and were performed twice. Task 8 was considered an advanced task and was performed only once. All tasks were performed in a Szabo–Berci–Sackier box trainer using Karl Storz laparoscopy instruments and a standard laparoscopy tower (KARL STORZ GmbH & Co. KG, Tuttlingen, Germany).

### Instruction mode

The verbal instructions were standardised for all participants and were delivered in a simple and standardized way. For the instruction with AR, the iSurgeon system [15–17] was used in addition to verbal instructions. Apart from that additional usage of the iSurgeon to telestrate hand gestures, there were no differences in the training between both instruction modalities. The iSurgeon system used an RGB-D camera (colour resolution: 1920 × 1080 pixels, 30 fps, depth resolution: 512 × 424 pixels, 30 fps) to detect the hands of the instructor and project them over the image on the laparoscopic screen. To operate it the instructor moved his hand beneath the sensor. Instructor and participant stood next to each other with enough space to not interfere with their movements. The instructions were provided by the same instructor throughout the study. Before the study, the instructor was introduced to the use of the iSurgeon and practiced it as well as the standardized verbal instructions until proficiency. The functional principle of the iSurgeon and the meaning of the verbal instructions were explained to the participants before the tasks were carried out.

### Eye tracking system

**Fig. 2:**
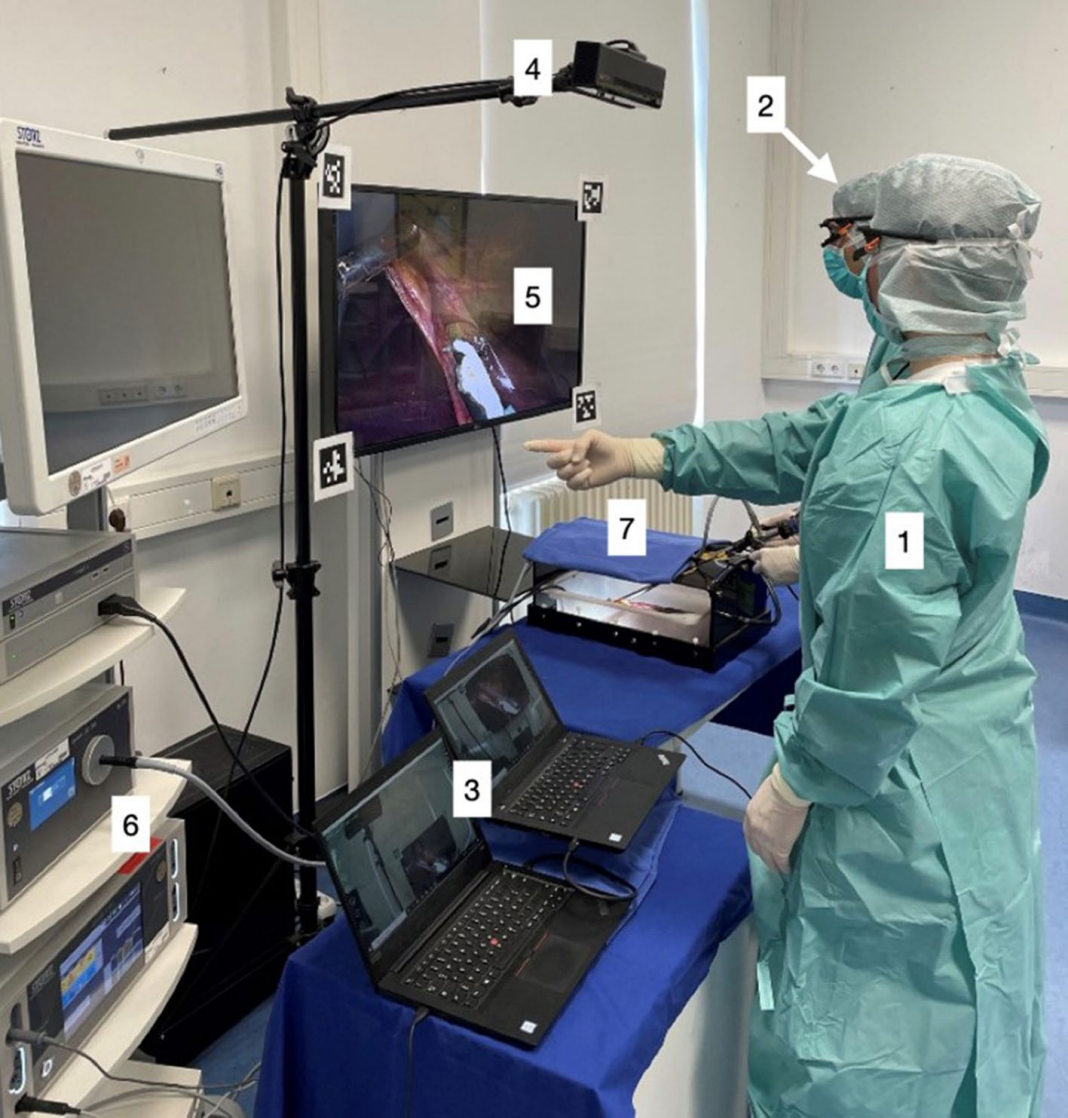
Instructor (1) and participant (2) wear Pupil Core eye-tracking glasses connected to separate laptops (3). When using AR, the instructor works with hand gestures detected by a sensor (4) to instruct the participant. The gestures are projected onto a screen (5) connected to a laparoscopic tower (6). All tasks are performed in a box trainer (7)

Pupil Core eye-tracking glasses (Pupil labs GmbH, Berlin, Germany) were used for the detection of both instructor’s and trainee’s eye movement (Fig. 2). The gazes were recorded using Pupil Capture software Version 1.23–4 (Pupil labs GmbH, Berlin, Germany). Further, the world cameras detected the environment in front of the instructor and the participant—in particular, the screen with the laparoscopic image. Overall, the gaze position, fixations, audio and the laparoscopic screen as a surface were recorded in a synchronized manner. The collected data were annotated and analysed with the Pupil Player software Version 1.23–4 (Pupil labs GmbH, Berlin, Germany) [24].

### Gaze metrics and performance assessment

The following gaze parameters were collected:

(i) Gaze latency. i.e. the time from instruction until the first fixation of the trainee’s gaze on a target area [19]
(ii) Gaze convergence, i.e. the absement, namely the integral of the gaze displacement, between the instruction and the first fixation of the trainee on the target area [18]
(iii) Collaborative gaze convergence, i.e. the absement of the instructor’s and trainee’s gaze over the time needed to merge over a target area [19]

For tasks 1–7, the performance was measured by the number of errors and time needed to complete the tasks. For the cholecystectomy, the global and task-specific Objective Structured Assessments of Technical Skills (OSATS) and the time needed to complete the tasks were used to measure the outcomes [25, 26]. The subjective workload of the participant was measured by a modified NASA Task Load Index (NASA-TLX) [27]. In addition, the blink rate was analysed as another marker for cognitive workload [28].

### Statistical analysis

Python 3 (Version 3.9.7, Python Software Foundation, Delaware, USA) with Pandas (Version 1.3.4, [29]) was used for data manipulation and Pingouin (Version 0.5.2, [30]) for statistical analysis. Within-group comparison was done with the Wilcoxon rank-sum test (tasks 1–7) and between-group comparison with the Mann–Whitney U test (task 8). These data were reported as medians and interquartile ratios. The effects of modality and task type to each metric were assessed with two-way repeated measures ANOVA and reported as *F*-values, *p*-values and *η*_p_^2^-values.

## Results

### Population characteristics

A total of 42 participants were screened. There were two dropouts due to personal reasons. The total number included in the study was 40. The study group characteristics can be found in Table 1. There was no relevant difference found in the population characteristics between both groups.

**Table 1:** Participants’ general characteristics

	Total	Group 1	Group 2
n, (%)	42 (100)	20 (50)	20 (50)
Age			
Mean (SD)		23.2 (3.5)	22,9 (2.9)
Gender			
Male, n (%)	20 (50)	11 (55)	9 (45)
Female, n (%)	20 (50)	9 (45)	11 (55)
Other, n (%)	0	0	0
Dropouts, n (%)	2 (4.7)	1 (50)	1 (50)
Academic Year		3.5 ± 0.6	3.4 ± 0.7

### Eye gaze outcomes

**Fig. 3:**
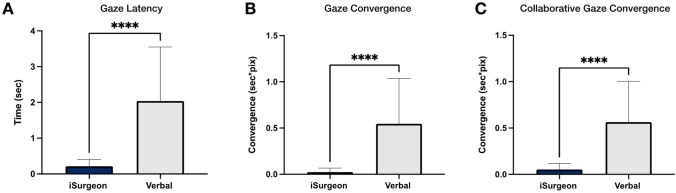
Eye gaze outcomes for tasks 1–7. **A** Gaze latency, gaze convergence. **B** and collaborative gaze convergence. **C** showed significantly lower results in the iSurgeon group, compared to the verbal group. ****p < 0.0001

Significant differences in all eye gaze parameters were observed in tasks 1–7 between both groups (iSurgeon vs. verbal). Gaze latency was significantly lower with AR, reaching approximately 0.21 ± 0.19 s for iSurgeon and 2.04 ± 1.51 s for the verbal only group (*F*(1,39) = 762.5, *p* < 0.01, *η*_p_^2^ = 0.95). Gaze convergence in iSurgeon group was also significantly lower at 0.02 ± 0.04 pixels*sec compared to 0.55 ± 0.49 pixels*sec in the verbal only group (*F*(1,39) = 482.8, *p* < 0.01, *η*_p_^2^ = 0.93). Collaborative gaze convergence also improved upon instruction with AR, reaching 0.05 ± 0.06 pixels*sec in the iSurgeon group and 0.56 ± 0.44 pixels*sec in the verbal only group (*F*(1,39) = 408.4, *p* < 0.01, *η*_p_^2^ = 0.91). (Fig. 3).

**Fig. 4:**
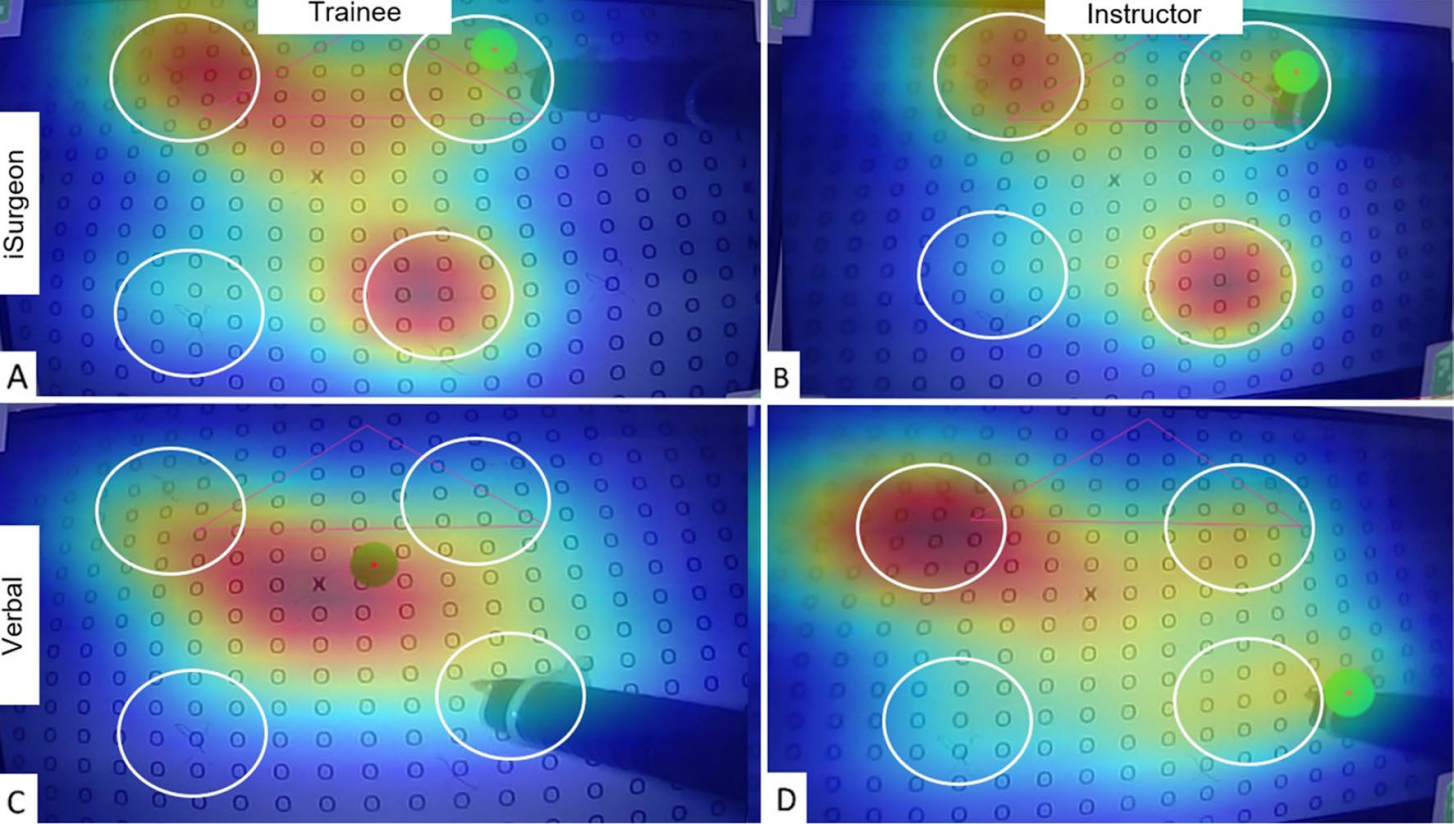
Exemplary heat map of eye gaze behaviour of the trainee (**A** + **C**) and instructor (**B** + **D**) upon verbal instruction (**C** + **D**) and with iSurgeon system (**A** + **B**). The target regions are circled in white. The red colour indicates a high number of gaze fixations (Color figure online)

The heatmaps of gaze fixations indicated that trainees focused on the target structures more precisely and had a higher overlap with the instructor’s gaze upon instruction with iSurgeon (Fig. 4).

### Performance and workload outcomes

**Fig. 5:**
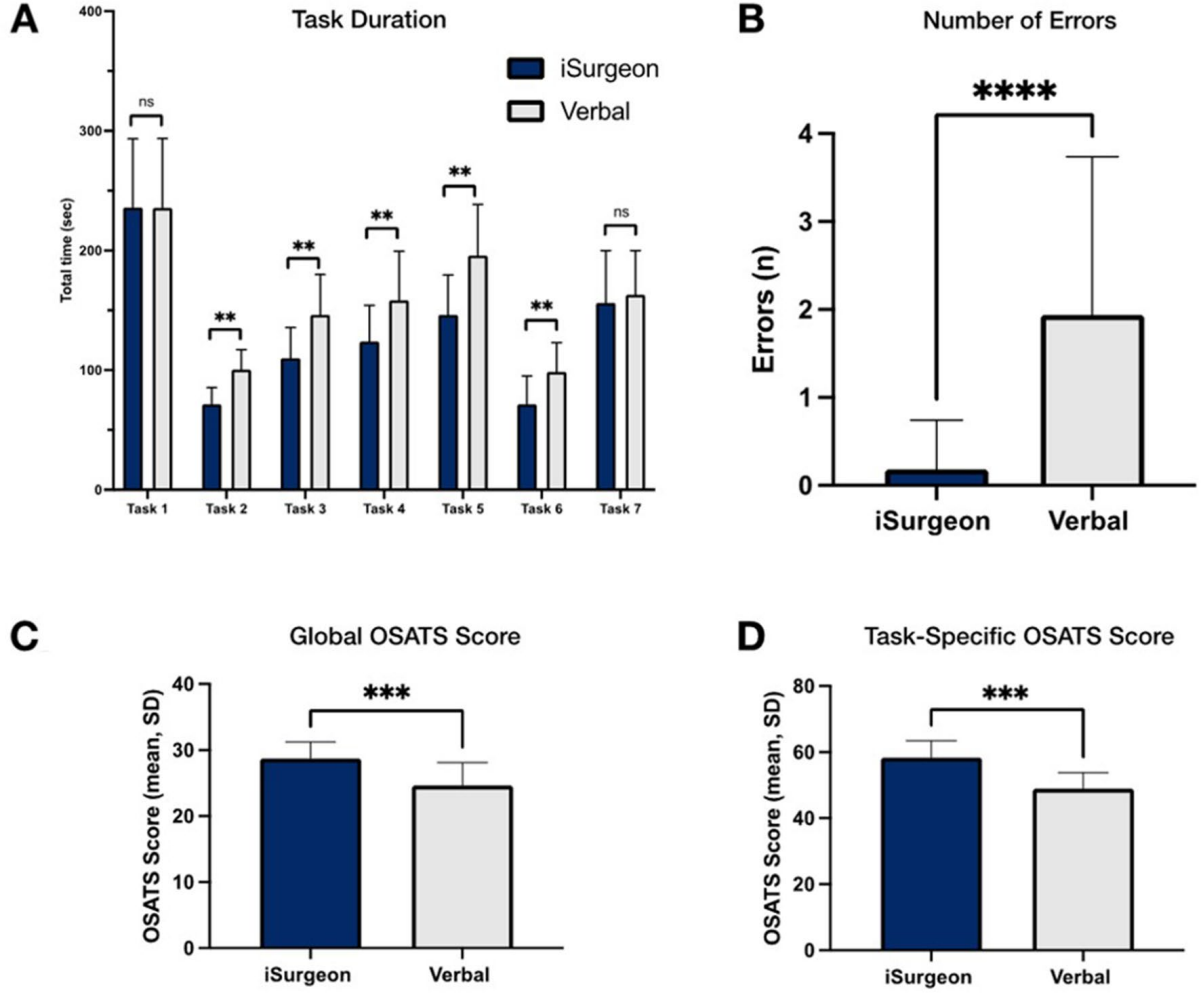
Performance outcomes. **A** Total task duration and **B** number of errors were measured for tasks 1–7. **C** + **D** The quality of the laparoscopic cholecystectomy was measured by global and task-specific OSATS. *ns*, not significant; *p < 0.05; **p < 0.01; ***p < 0.001; ****p < 0.0001

Using the AR system for instruction resulted in a lower number of errors (tasks 1–7; 0.18 ± 0.56 vs. 1.94 ± 1.80, *F*(1,39) = 433.5, *p* < 0.01, *η*_p_^2^ = 0.92) and faster completion time (tasks 1–7; 118 ± 73 vs. 148 ± 81.5 s, *F*(1,39) = 97.7, *p* < 0.01, *η*_p_^2^ = 0.71) compared to verbal instruction only (Fig. 5). No significant difference in task duration was shown for task 8 (4781 ± 1257 vs. 5.024 ± 1447 s, *F*(1) < 0.01, *p* = 0.98, *η*_p_^2^ < 0.01). Also, the score ratings for laparoscopic cholecystectomy (task 8) were significantly higher in the iSurgeon group, resulting in higher mean global OSATS of 29 ± 2.5 vs. 25 ± 5.5, (*p* < 0.01) and mean task-specific OSATS of 60 ± 3 vs. 50 ± 6 (*p* < 0.01, Fig. 5).

**Fig. 6:**
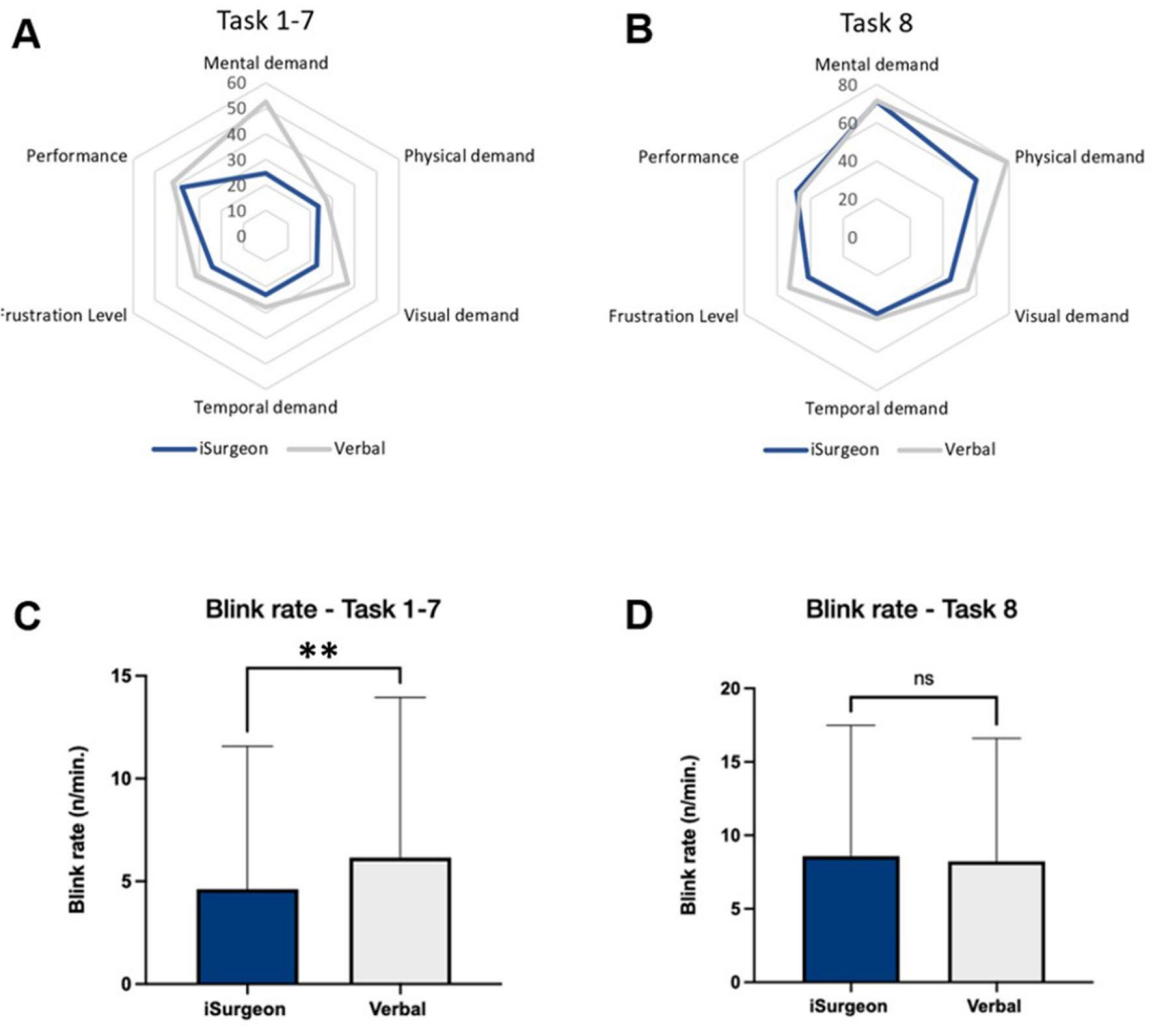
Cognitive workload of the trainee measured by **A** + **B** NASA-TLX questionnaire and **C** + **D** blink rate. *ns*, not significant; *p < 0.05

Instruction with the AR system resulted in significantly lower cognitive workload in the NASA-TLX in tasks 1–7 (50 ± 21 vs. 56 ± 22, p < 0.01), whereas it was not significantly lower during laparoscopic cholecystectomy (28 ± 14 vs. 37 ± 15, *p* = 0.12) (Fig. 6). Throughout the basic tasks (tasks 1–7) the blink rate was lower upon instruction with iSurgeon (1.8 ± 5.2 vs. 3.4 ± 6.2, *F*(1,39) = 39.62, *p* < 0.01, *η*_p_^2^ = 0.50) while no significant differences were found in task 8 (4.7 ± 9.1 vs. 5.0 ± 7.9, *F*(1) = 0.05, *p* = 0.83, *η*_p_^2^ < 0.01, Fig. 6).

## Discussion

In the present study, telestration with augmented reality using the iSurgeon system successfully improved not only the trainee’s gaze behaviour but also resulted in improved laparoscopic performance. Additionally, the cognitive workload was reduced in most of the tasks.

The Trainee’s gaze behaviour was improved by reducing the gaze latency and improving the gaze convergence and the collaborative gaze convergence in the surgical tasks in the present study using the augmented reality telestration system as compared to only using verbal guidance in laparoscopic surgical training. The lower gaze latency with the iSurgeon indicates a lower time from instruction to fixation on targets [18]. This could have helped the trainee to identify the targets faster. The faster target identification may have led to the higher gaze convergence, which indicates a higher convergence of the participants’ gaze and the target areas [18]. The higher collaborative gaze convergence indicates that the gaze of trainer and trainee converged more during the task [19]. One important purpose of telestration systems like the iSurgeon is to help a novice to see what an expert sees [15–17]. The higher collaborative gaze convergence indicates that this purpose of the iSurgeon was achieved in the study. In the last years, an increasing number of studies observing the gaze behaviour of surgeons in MIS and surgical training have been published [12, 18–22]. Several studies showed that more experienced surgeons have a higher fixation rate (median (IQR) 1.86 (0.3) vs 0.96 (0.3); *p* = 0.006) and a higher dwell time (median (IQR) 792 (159) vs 469 (109) s; *p* = 0.028) than novices while performing more complex laparoscopic procedures, e.g. inguinal hernia repair [20]. Further, it has been shown that experts look predominantly at target structures (*r* = 0.655, *p* < 0.05) and less at non-essential structures than novices (*r* = − 0.619, *p* < 0.05) [21] and disengage their gaze faster from the previous subtask (mean(SD) expert surgeons: − 1(93) ms, intermediate surgeons: − 189(160) ms, novices: − 296(179) ms) while performing cholecystectomies [22]. However, novices’ gazes became more focused and less scattered upon instruction with AR pointers [12]. Further, the time between instruction and the gaze on a target (gaze latency) decreased by 48% and the convergence of gaze and target over time (gaze convergence) improved by 33% while being instructed with AR compared to verbal instruction [18, 19]. But only gaze latency, gaze convergence and collaborative gaze convergence seemed to correlate with a better performance during training, especially with lower error rates and less time needed to complete basic laparoscopic tasks [18, 19]. The results in the present study matched with previous studies analysing gaze latency, gaze convergence and collaborative gaze convergence, as it also showed an improvement with telestration. This suggests that AR-based telestration works by means of guiding the trainees’ gazes and is thus a promising tool to improve gaze behaviour in laparoscopic training.

The surgical performance of the participants in the present study was improved with the iSurgeon through lower error rates, higher performance scores and reduced times in most of the tasks. The error rate was reduced in all the tasks which indicates that AR telestration led to fewer misconceptions and improved the quality of the tasks. Also, the achieved points in the global and task-specific OSATS scores indicate an improved quality as compared to only verbal guidance. The higher global OSATS score indicates an improved general surgical performance during task 8. The higher task-specific OSATS score indicates a better performance of the cholecystectomy for the use of telestration with AR [31, 32]. A reason for the improved quality of the performances in the tasks might have been improved communication through the iSurgeon [16]. Improving communication not only in training but also in the real operating theatre is essential. Because multiple studies have shown that unclear communication and misunderstandings can lead to intraoperative mistakes and cause complications [33, 34]. Therefore, it is essential to try to improve intraoperative communication—one possibility for that could be telestration with AR [16]. The time efficiency was improved in the basic tasks (tasks 1–7) in the present study with AR when comparing them combined but showed no difference in task 8. The improvement of the time efficiency in the basic tasks could have been achieved through faster recognition of the target structures by the participants. A reason why the time was not reduced in the cholecystectomy (task 8) could have been the complexity of the task. Cholecystectomy is a rather complex task for laparoscopically naïve medical students and this could explain the similar task times, albeit better performance in the AR group compared to the control. The clearer instructions with AR at key steps may have led to a better quality of the tasks by preventing mistakes. Analysing the task duration is still important because transferred to the operating theatre longer operation times can increase the risk of complications like wound infections, thromboses and cardiopulmonary failure [35, 36]. The results of this study indicate that instructing trainees in laparoscopic training with AR can improve the quality of their performance and reduce the task time in most of the tasks. Also, the performance in more complex procedures like cholecystectomy was influenced by AR through improving the performance compared to verbal instructions. Hence, AR telestration may improve the quality and efficiency of surgical training. Translation to improved clinical outcomes will have to be shown in future studies.

The cognitive workload was improved with AR compared to verbal instructions by lowering the NASA-TLX and the blink rate in most of the tasks. Both, the score in the NASA-TLX and the blink rate were lower during instruction with AR in tasks 1–7 combined but showed no significant difference in task 8. The lack of workload reduction in task 8 seems to be in concordance with the lacking time difference between both instruction modalities in task 8. The reason for that might also have been that the task was too complex for the inexperienced participants causing the iSurgeon to not have a significant effect on the already very high workload by the task itself. However, the lower score in the NASA-TLX in the basic tasks indicates a reduced subjective workload [27] The lower blink rate in the basic tasks indicates a reduced objectively measured workload in those tasks [28, 37, 38]. Measuring the workload during training and in the operating theatre is an established way to analyse surgical performances [39] [40]. Decreasing the workload of surgeons is especially important in the real operating theatre. It has been shown that an elevated workload can cause higher error rates and worse intraoperative performances of surgeons [41]. In this study, AR telestration was able to reduce both the subjective and objective workload of the participants in most of the tasks. Therefore, AR telestration may be a way to reduce workload during surgical training.

The study has some limitations. The data annotation of the eye gaze data could not be blinded due to technical reasons, i.e. the projected iSurgeon hand of the instructor was visible throughout the analysis process. To limit a possible detection bias caused by the unblinded data annotation annotating with strict and objective standards and through only having one person who annotated all the data were performed. Yet, the blink rate could not be influenced by the annotator and was determined directly from the recorded and exported data. Another limitation of the study is the transferability into the real operating theatre. It is unclear if the results observed in the tasks in the box trainer and the cadaveric cholecystectomy would also be observed during real surgical procedures. However, the gaze behaviour was analysed during multiple and different tasks to mimic as many different aspects of surgical procedures as possible. Additionally, the participants were not surgeons but laparoscopically naïve medical students. This may reduce the transferability of the results on surgeons but has the advantage of having a homogenous study population.

In the present study, telestration with AR led to an improved surgical performance in training. AR improved the gaze behaviour by reducing the time from instruction to target fixation and improving the convergence of the participants’ gazes with target areas as well as with the instructor’s gaze. Additionally, the quality and time efficiency was improved and the workload was reduced in most of the tasks using telestration with AR with the iSurgeon system. These results suggest that AR systems may be effective training tools by adjusting trainee’s gaze behaviour to instructor’s, which may result in better overall performances and trainee’s comfort while performing tasks. Hence, it may be a promising tool to improve surgical training. If these results could transfer to the operating theatre, intraoperative communication and gaze behaviour could be improved, which may lead to fewer misunderstandings, errors, complications and reduce intraoperative time. Future studies are needed to assess implementation possibilities into clinical practice and the effects of AR telestration in different training situations as well as in the operating theatre.

## Supplementary Information

Below is the link to the electronic supplementary material.

Supplementary file1 (JPG 141 kb)—**Supplementary Fig. 1** Instructor’s hand is displayed on the laparoscopic screen during the laparoscopic task. **A** PEG transfer, **B** Marking Circles, **C** Needle Parkour, **D** Grabbing and transferring vessel loops **E** Unravelling silicone small intestine, **F** Suture ligations of blood vessel in a silicone model, **G** Picking up felt cloth, **H** Cholecystectomy in a cadaveric poricine liver model

Supplementary file2 (DOCX 13 kb)—**Supplementary Table 1** Laparoscopic tasks
