# Supplementary figures and images for: Telestration with augmented reality improves surgical performance through gaze guidance
Source: Surg Endosc. 2023 Jan 6;37(5):3557–66. doi: 10.1007/s00464-022-09859-7 (PMC10156835; doi:10.1007/s00464-022-09859-7)

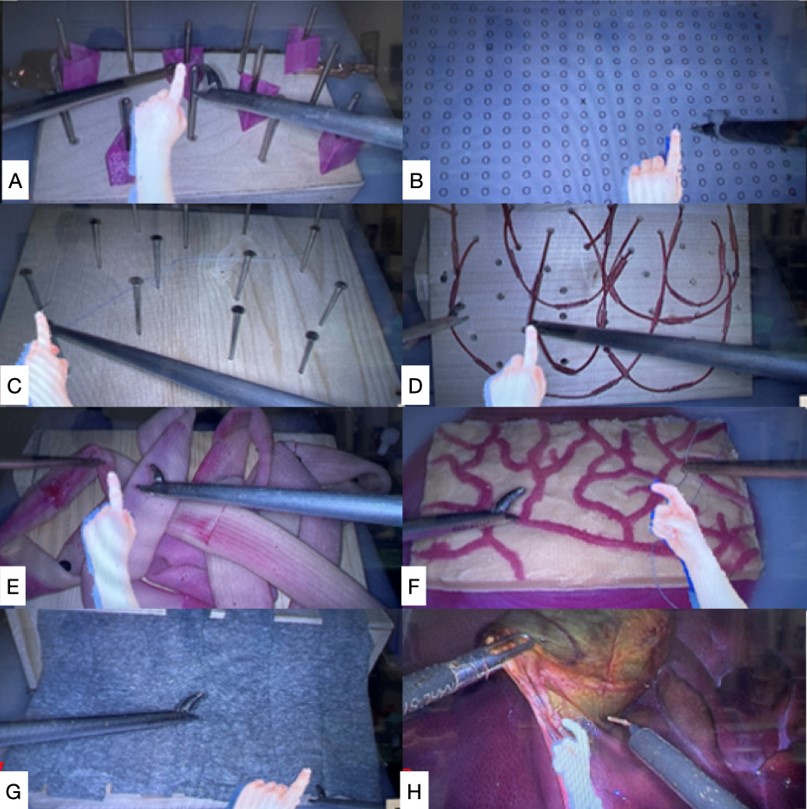

Supplement: Supplementary file 1 — Supplementary file1 (JPG 141 kb)—Supplementary Fig. 1 Instructor’s hand is displayed on the laparoscopic screen during the laparoscopic task. A PEG transfer, B Marking Circles, C Needle Parkour, D Grabbing and transferring vessel loops E Unravelling silicone small intestine, F Suture ligations of blood vessel in a silicone model, G Picking up felt cloth, H Cholecystectomy in a cadaveric poricine liver model [file 464_2022_9859_MOESM1_ESM.jpg]
