# Supplementary material for: Telestration with augmented reality improves surgical performance through gaze guidance
Source: Surg Endosc. 2023 Jan 6;37(5):3557–66. doi: 10.1007/s00464-022-09859-7 (PMC10156835; doi:10.1007/s00464-022-09859-7)
Supplement: Supplementary file 2 — Supplementary file2 (DOCX 13 kb)—Supplementary Table 1 Laparoscopic tasks [file 464_2022_9859_MOESM2_ESM.docx]

| Task | Explanation |
| --- | --- |
| 1. PEG transfer | The participants were guided by the instructor to pick up PEGs from one side of a wooden board and to place them on iron sticks on the other side of a wooden board in a specific order and position. |
| 2. Marking Circles | The participants were instructed to mark specific circles on a sheet of paper with a printed grid of circles on it. |
| 3. Needle Parkour | The participants held a needle with thread in a needle holder and followed the instructions in which order to move the needle through a parkour consisting of nails in a wooden board. |
| 4. Picking up vessel loops | The participants were instructed to grab silicone vessel loops attached to a wooden board in a specific order and position and move them over the edge of the board. |
| 5. Unraveling silicone small intestine | The participants were instructed to grab certain loops of a silicone small intestinal convolute in a specific order to unravel it and bring it into a set configuration. |
| 6. Suture ligations of blood vessel silicone model | The participants were instructed to perform suture ligations of blood vessels in a silicone model in definite positions using a needle with thread. |
| 7. Picking up felt cloth | The participants had to pick up pieces of felt cloth in a specific order and lay them over the edge of the wooden construction where they were attached to. |
| 8. Cholecystectomy | Cholecystectomy of a cadaveric porcine gallbladder. This task was divided into three steps: 1. Preparation of the cystic artery and duct, 2. clipping and cutting of the cystic artery and duct and 3. preparation of the gallbladder from the liver. |
